# Seasonal climate manipulations have only minor effects on litter decomposition rates and N dynamics but strong effects on litter P dynamics of sub-arctic bog species

**DOI:** 10.1007/s00442-012-2330-z

**Published:** 2012-04-21

**Authors:** R. Aerts, T. V. Callaghan, E. Dorrepaal, R. S. P. van Logtestijn, J. H. C. Cornelissen

**Affiliations:** 1 Systems Ecology, Department of Ecological Science, VU University Amsterdam, De Boelelaan 1087, 1081 HV Amsterdam, The Netherlands; 2 Abisko Scientific Research Station, Royal Swedish Academy of Sciences, 981 07 Abisko, Sweden; 3 Department of Animal and Plant Sciences, Sheffield Centre for Arctic Ecology, The University, Sheffield, S10 2TN UK; 4 Present Address: Climate Impacts Research Centre, Umeå University, 981 07 Abisko, Sweden

**Keywords:** Climate warming, Immobilization, Nutrient limitation, Nutrient mineralization, Phosphorus release

## Abstract

Litter decomposition and nutrient mineralization in high-latitude peatlands are constrained by low temperatures. So far, little is known about the effects of seasonal components of climate change (higher spring and summer temperatures, more snow which leads to higher winter soil temperatures) on these processes. In a 4-year field experiment, we manipulated these seasonal components in a sub-arctic bog and studied the effects on the decomposition and N and P dynamics of leaf litter of *Calamagrostis lapponica*, *Betula nana*, and *Rubus chamaemorus*, incubated both in a common ambient environment and in the treatment plots. Mass loss in the controls increased in the order *Calamagrostis* < *Betula* < *Rubus*. After 4 years, overall mass loss in the climate-treatment plots was 10 % higher compared to the ambient incubation environment. Litter chemistry showed within each incubation environment only a few and species-specific responses. Compared to the interspecific differences, they resulted in only moderate climate treatment effects on mass loss and these differed among seasons and species. Neither N nor P mineralization in the litter were affected by the incubation environment. Remarkably, for all species, no net N mineralization had occurred in any of the treatments during 4 years. Species differed in P-release patterns, and summer warming strongly stimulated P release for all species. Thus, moderate changes in summer temperatures and/or winter snow addition have limited effects on litter decomposition rates and N dynamics, but summer warming does stimulate litter P release. As a result, N-limitation of plant growth in this sub-arctic bog may be sustained or even further promoted.

## Introduction

Northern peatlands store about 4.5 × 10^17^ g (=450 Pg) of carbon which is about 30 % of the total global soil C pool. Currently, they serve as a net sink for atmospheric carbon with an estimated accumulation rate of 0.07–0.08 Pg C year^−1^ (Gorham 1991). The carbon accumulation rate of these peatlands is determined more by low decomposition rates of plant litter and soil organic matter (SOM) than by high primary production. These low decomposition rates are caused by low temperatures, water-logged (anoxic) and acidic site conditions, low nutrient concentrations in plant litter, and/or high concentrations of secondary compounds such as lignin and phenolics (Robinson 2002; Aerts et al. 2006a).

It is predicted that climate change due to greenhouse gas emissions will lead to an increase of the mean global temperature by 2.5–7.0 °C in the next 50–100 years, with above-average increases at high-latitude and high-altitude sites (IPCC 2007). Climatic effects on litter decomposition can operate both directly and indirectly (Aerts 2006). At short timescales, they can operate directly through changes in soil temperature and soil moisture that alter rates of litter mass loss due to the high sensitivity of biological processes to temperature and water availability. At longer timescales, they can operate indirectly through the effects of warming on plant litter quality. Such effects can be caused either through phenotypic responses of the species in the community or, on longer timescales, through the effects on plant community structure and subsequent effects on plant litter quality (Cornelissen et al. 2007). The phenotypic response of plant litter chemistry to summer warming usually involves a slight reduction (5–15 % lower than controls) of litter N concentration, although there is substantial interspecific variation in the response (cf. Dormann and Woodin 2002; Aerts et al. 2006a, 2009; Dorrepaal et al. 2006). Due to the positive relation between litter N content and decomposability, this phenotypic response of litter chemistry may in the short term lead to lower decomposition rates. However, longer-term changes to leaf litter decomposition will be driven primarily by both direct warming effects and concomitant changes in plant growth form composition, and to a much lesser extent by phenotypic responses in leaf litter quality (Cornelissen et al. 2007). Thus, the overall effect of higher temperatures is determined by the balance between direct temperature effects and indirect effects on litter chemistry and/or species composition.

The direct temperature effect may not only lead to higher decomposition rates but may also speed up the nutrient mineralization rates in the litter and the soil organic matter. A re-analysis of experimental warming studies (Rustad et al. 2001) for nine tundra sites showed that heating over at least 3 years increased net soil N-mineralization by about 70 % (Aerts et al. 2006b). This higher N availability may also lead to higher leaf N concentrations and thereby higher leaf litter decomposition rates. However, in a N-fertilization experiment in a sub-arctic bog, we found both positive, negative, and neutral effects of N addition on leaf litter decomposition (Aerts et al. 2006a).

A further complication in the prediction of climate change effects on litter decomposition and nutrient mineralization rates is that climate change in cold biomes not only involves higher summer temperatures but also warmer springs and more winter precipitation (IPCC 2007). So far, little is known about the response of litter decomposition and nutrient mineralization rates to these seasonal components of climate change in cold biomes (Aerts et al. 2006b). Earlier studies have shown that winter decomposition can contribute substantially (up to 20 %) to annual mass loss rates if litter fall occurs in the autumn (Moore 1983, 1984; Hobbie and Chapin 1996). However, there is only very little information on the effect of more snow cover on decomposition and nutrient mineralization rates. Given the observed relatively high winter decomposition rates, it might be expected that more winter snow cover (and thus less cold soil temperatures under the snow and earlier soil thawing in spring) can lead to higher decomposition and nutrient mineralization rates. However, this depends on the thickness of the snow cover and the difference in temperature between air and the location at where the litter decomposes (the soil surface). This hypothesized higher decomposition in response to more snow is supported by data of Wallenstein et al. (2009), who found that potential soil enzyme activities in arctic tundra soils were highest at the end of winter, while soils were frozen. Moreover, they found that soil enzyme pools responded stronger to temperature change (higher Q_10_ values) at the end of winter than during the summer.

These observations raise the question how the observed and predicted increase in temperature and winter precipitation due to global change will affect litter decomposition and nutrient mineralization rates of dominant plant species, and thus the cycling of carbon and nutrients in high-latitude ecosystems. We hypothesized that (1) differences in litter chemistry between dominant species are much stronger drivers of litter decomposition rates than phenotypic changes of litter chemistry in response to climate warming; and (2) both summer warming and spring warming, and to a lesser extent increased winter snow cover, increase litter mass loss and litter N and P mineralization rates.

To test these hypotheses, we performed a 4-year field experiment in which we investigated the effects of experimental seasonal climate manipulations on decomposition and N and P dynamics of leaf litter of three dominant species of vascular sub-arctic bog vegetation in northern Sweden. The species were *Calamagrostis lapponica* (Wahlenb.) Hartm. (graminoid), *Betula nana* L. (woody deciduous), and *Rubus chamaemorus* L. (perennial herb). We disentangled the direct thermokinetic effects of the climate manipulations and the indirect effects (through phenotypic changes in litter chemistry only) by incubating litter that was formed within each treatment within the treatment plots, and by incubating litter that was formed within all treatments under ambient conditions, respectively.

## Materials and methods

### Study site

The study was performed on a sub-arctic, north-facing sloping bog near the Abisko Scientific Research Station in northern Sweden (68°21′N,18°49′E). The site is located at an altitude of about 340 m a.s.l. and is bordered by Lake Torneträsk to the north and a mosaic of birch forest and small mires to the south. Annual precipitation amounts to 303 mm year^−1^ of which about 50 % falls in the form of snow (1913–2006; Johansson et al. 2008). Snow thickness in winter at the site is about 15 cm. This relatively shallow layer is not only caused by the relatively low amount of snow fall but also due to the very wind-exposed character of the site, which results in much of the snow being blown away to the adjacent lake. The mean summer temperature is 7 °C and the mean winter temperature is −6 °C. The length of the growing season is about 130 days (Karlsson and Callaghan 1996). The moss component of the bog is dominated by *Sphagnum fuscum* (Schimp.) H.Klinggr. The cover of vascular plants is about 25 % and mainly consists of the evergreen dwarf shrubs *Empetrum hermaphroditum* Hagerup and *Andromeda polifolia* L., the deciduous dwarf shrubs *Betula nana* and *Vaccinium uliginosum* L., the grass *Calamagrostis lapponica*, and the forb *Rubus chamaemorus* (Keuper et al. 2011).

### Experimental design of the climatic change experiment

Currently, there is much uncertainty about future climates at high-latitude sites. Most climate models agree that summers will be warmer but also that winters may become wetter (more snow) and that the summer season will be extended (warmer spring period) (IPCC 2007). However, it is not clear if all these changes will occur simultaneously. Therefore, in June 2000, we started a long-term experiment incorporating six different experimentally imposed climatic scenarios on sub-arctic bog vegetation and soil in northern Sweden (Dorrepaal et al. 2004; Aerts et al. 2004). These scenarios (Table 1) consisted of a mixture of summer warming (June–September), additional snow accumulation in winter (October–late April), and spring warming (late April–May). Due to practical constraints, we were not able to lay out a full factorial design with these three factors with sufficient replication. Therefore, we chose those combinations that were in our opinion among the most likely representatives of future climate scenarios. The experiment had a blocked design with five replications per treatment.

**Table 1:** Climate treatments used in the experiment

Treatment		Summer	Winter	Spring
Number	Code			
1	AAA	A	A	A
2	ASA	A	S	A
3	ASW	A	S	W
4	WAA	W	A	A
5	WSA	W	S	A
6	WSW	W	S	W

*A* ambient, *W* warming, *S* (passive) snow accumulation

Spring and summer warming were established by passive warming using a modified, larger version of the ITEX-open-top chambers (OTCs; see Marion et al. 1997). Our hexagonal OTCs were 50 cm high, had a diameter of 1.6–1.8 m at the top and 2.2–2.5 m at the base, and were made of transparent polycarbonate (Makro Life, Arlaplast, Sweden). We chose larger OTCs to reduce edge effects from reduced precipitation below the OTC panels and clonal connections beyond the plots. Increased winter snow accumulation was achieved by leaving the OTCs in place to serve as passive snow traps.

The annual summer warming treatments (treatments 4–6) involved placement of an OTC on a plot from early June until the end of September (end of each growing season), when OTC positions were changed to prepare for the winter treatments. In late April, the OTCs were removed from snow accumulation treatments without spring warming, and excess snow above the ambient level was removed (treatments 2 and 5), but the OTCs were left in place for the spring warming treatments (treatments 3 and 6). At the beginning of June, the OTCs were moved to the summer positions again (treatments 4–6).

We found that our climate manipulations had moderate but significant and realistic effects on air and soil temperatures (details in Dorrepaal et al. 2004, 2009): in winter, the OTCs increased the snow thickness two-fold from about 15–30 cm, resulting in 0.5–2.8 °C higher average temperatures at 5 cm above the soil surface (so in the snow) and 1.7 °C higher soil temperatures at 5 cm depth. Spring warming increased air temperatures in the OTCs by 0.7–1.2 °C, whereas summer warming had a maximum effect of 0.9 °C. The data available so far showed no indications of effects of the treatments on soil moisture in the central part of the OTCs, because vapor pressure deficit was not affected by the OTCs.

### Litter bag study

We determined litter decomposition rates of three dominant vascular species at our experimental site: *Betula nana*, *Calamagrostis lapponica*, and* Rubus chamaemorus*. Litter of these species was collected in September in each plot, after 4 years of treatment. It was not possible to collect litter in sufficient amounts for the other species due to their low cover or, as was the case for the evergreens, the fact that senescing leaves were overgrown by *Sphagnum fuscum* (cf. Dorrepaal et al. 2006; Keuper et al. 2011) which made it impossible to collect them without completely destroying our plots.

Litter decomposition rates were determined in the field using the litter bag method. The litter from each plot was used to prepare litter bags by putting 0.300 g (*Rubus* 0.600 g) of air-dried litter in polyethylene litter bags with a mesh-width of 0.9 mm (*Calamagrostis* 0.3 mm). We chose this relatively small mesh width to reduce the chance that *Calamagrostis* leaves or fragmented litter of other species would be lost from the litter bags. As decomposition in sub-arctic areas is controlled most strongly by micro-organisms and micro-invertebrates and not by the larger detritivores (Swift et al. 1979; Makkonen et al. 2011), the most relevant organisms could access the litter samples in the bags. Ratios between air-drid mass and oven-drid mass were determined on ten sub-samples of 0.500 g of air-dried litter of each species after drying for 48 h at 70 °C.

For each species, two litter bags were incubated in the plot where the litter was collected (‘climate treatment incubation’), whereas two other litter bags were transplanted to a common ‘ambient’ incubation environment outside the experiment but with similar vegetation and exposure (‘ambient incubation’). In this way, we were able to differentiate between the overall and indirect treatment effects (see “Introduction”). In the OTC plots, the litter bags were placed horizontally on the soil surface in the central part of the OTCs so that they received the normal amount of precipitation. Litter bags of each species, treatment and incubation environment were collected after 2 and 4 years. After retrieval, extraneous litter, soil particles, and roots were removed from the litter bags and the remaining dry mass of the litter was determined after drying for 48 h at 70 °C.

Nutrient concentrations in the initial litter (determined for each plot separately, so *n* = 5 for each treatment) and in the remaining litter after 4 years of decomposition were determined by standard methods. Total C and N concentration were determined by dry combustion of ground plant material with a NA1500 elemental analyser (Carlo Erba, Rodana, Italy). Total P concentrations were determined for all litter samples by digesting ground leaf material in 37 % HCl:65 % HNO_3_ (1:4, v/v). Phosphorus concentration was measured colorimetrically at 880 nm after reaction with molybdenum blue.

### Statistical analysis

Prior to statistical analysis, data were tested for homogeneity of variances using Levene’s test and transformed where appropriate. Due to the non-orthogonal design of our experiment, we could not include interactions between the main factors in our analysis of leaf nutritional variables and mass loss. However, these interactions may be very important for a proper analysis of our results. Therefore, we chose a stepwise analysis in which we first considered the spring treatment as part of the winter treatments and then performed a full factorial four-way ANOVA with species, incubation environment (ambient vs. treatment), summer treatment (see Table 1), and winter treatment (see Table 1) as the main factors and with all possible interactions (as was done in Aerts et al. 2004). Next, we followed our original design, and analyzed the data for each species separately with the spring treatment separate, and analyzed our data with a three-way ANOVA with the three seasons (summer, winter, spring treatments) as independent factors. As this design was non-orthogonal (see Table 1), we analyzed main effects only and not the interactions (as in Aerts et al. 2004).

## Results

### Treatment effects on initial litter chemistry

**Fig. 1:**
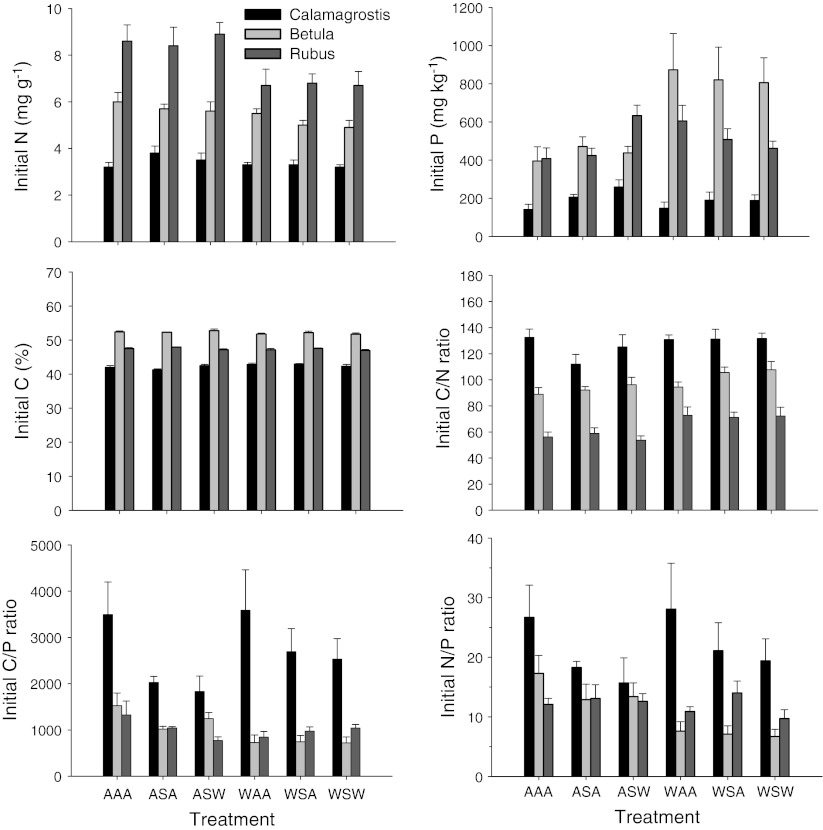
Initial nutrient parameters of leaf litter of three sub-arctic bog species in relation to climate treatments (see Table 1). Data are means ± SE (*n* = 5)

The three species differed strongly in initial litter N, P, and C concentration and in C/N, C/P, and N/P ratio (Fig. 1; Table 2). In the control treatment, initial N concentration varied about threefold among species in the order *Calamagrostis* < *Betula* < *Rubus*. Litter C concentrations in the controls varied between 42.0 ± 0.5 % (*Calamagrostis*) and 52.4 ± 0.3 % (*Betula*) with an intermediate value for *Rubus* (47.5 ± 0.3 %). Although these differences were highly significant (Table 2), the relative differences were small so that the pattern in litter C/N ratio mirrored that in litter N concentration.

**Table 2:** Results of three-way ANOVAs for leaf litter nutrient parameters as dependent on species and on summer and winter treatments (see Table 1)

	N	P	C	C/N	C/P	N/P	N remaining	P remaining
Species (*df* = 2)	164.4***	115.6***	1,043***	195.9***	68.2***	25.1***	1.4	39.7***
Summer treatment (*df* = 1)	22.6***	4.2*	0.1	23.4***	4.1*	8.0**	0.2	16.7***
Winter treatment (*df* = 2)	0.1	0.4	0.2	0.1	2.9	2.2	1.0	3.8*
Species × summer (*df* = 2)	7.0**	12.0***	5.6**	3.4*	8.2**	8.8***	1.6	8.1**
Species × winter (*df* = 4)	0.7	0.3	1.0	1.3	1.2	0.9	2.4	3.2*
Summer × winter (*df* = 2)	0.2	0.4	2.1	0.4	2.6	0.2	0.5	2.1
Species × summer × winter (*df* = 4)	0.2	0.5	0.9	0.5	0.5	0.4	0.4	0.8

The *F* values for the main effects and their interactions are presented, together with their level of significance. For all variables, error degrees of freedom (*df*) = 71

* P < 0.05, ** *P* < 0.01, *** *P* < 0.0001

The summer treatment significantly affected litter N concentration, but not for all species, as is shown by the significant species × summer interaction (Table 2). Summer warming reduced the litter N concentration of *Betula* with only 11 % (relative to the control) and that of *Rubus* with 26 %, whereas there was no effect on *Calamagrostis* (Fig. 1; Table 3). Although there was no significant overall summer treatment effect on litter C concentration, there was a significant species × summer interaction (Table 2), indicating that species litter C concentrations responded in an opposite manner to the summer treatment, but the relative responses were very small. As a result, the response of litter C/N ratios to the treatments mirrored that of litter N concentration (Fig. 1; Table 3).

**Table 3:** Results of three-way ANOVAs for leaf litter nutrient parameters as dependent on summer, winter, and spring treatments (see Table 1)

	N	P	C	C/N	C/P	N/P	N remaining	P remaining
*Calamagrostis lapponica*								
Summer	1.5	1.1	3.6	2.2	1.3	0.4	0.5	1.7
Winter	2.5	3.6	0.2	2.2	3.6	1.9	1.6	1.1
Spring	0.8	0.3	0.2	0.9	0.3	0.6	1.4	5.1*
*Betula nana*								
Summer	7.9**	19.7***	4.2*	7.3*	17.4***	26.1***	0.2	20.6***
Winter	2.3	0.3	0.2	2.6	0.6	0.8	1.1	0.6
Spring	0.1	0.1	0.1	0.3	0.2	0.1	6.1*	1.4
*Rubus chamaemorus*								
Summer	15.8***	2.3	2.1	16.5***	0.4	0.6	2.3	14.8**
Winter	0.1	0.8	2.6	0.1	0.1	0.6	0.0	0.1
Spring	0.2	2.4	6.7*	0.3	0.8	1.4	0.8	1.6

The *F* values for the main effects are presented, together with their level of significance. For all variables, error *df* = 26

* P < 0.05, ** *P* < 0.01, *** P < 0.0001

In the controls, the P concentration of *Calamagrostis* litter was the lowest of all three species, but those of *Betula* and *Rubus* were equal (Fig. 1; Table 2). As with N, the C/P ratio mirrored that of the P concentration due to the relatively constant C concentration among species. The summer treatment significantly affected litter P concentration, but not for all species, as is shown by the significant species × summer interaction (Table 2). Summer warming increased P concentration of *Betula* with 82 % (relative to the control), but there was no significant effect on the other species. Also, for P, the response of litter C/P ratios to the treatments mirrored that of litter P concentration (Fig. 1; Table 3).

Litter N/P ratios decreased in the order *Calamagrostis* > *Betula* > *Rubus* (Fig. 1; Table 2). The summer treatment reduced the N/P ratio, but this was species-specific, as it only occurred in *Betula* (Table 3). This was mainly due to the strong increase in litter P in response to summer warming in this species.

### Incubation environment and treatment effects on litter mass loss

**Fig. 2:**
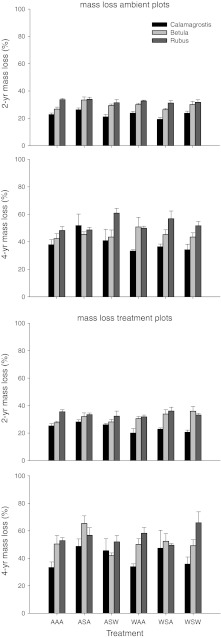
Mass loss (%) of leaf litter of three sub-arctic bog species after 2 and 4 years of incubation in relation to climate treatments (see Table 1). Litters from the various treatments were incubated in a common, ambient environment (*ambient plots*) or in the treatments from which the litter originated (*treatment plots*). Data are means ± SE (*n* = 5)

Mass loss rates in this sub-arctic peatland were relatively low and differed significantly among species with on average the lowest mass loss in *Calamagrostis*, intermediate mass loss in *Betula*, and the highest mass loss in *Rubus* (Fig. 2; Table 4). After 4 years, mass loss in the controls of the treatment plots ranged from 33.3 ± 4.0 % mass loss in *Calamagrostis* to 52.8 ± 2.1 % in *Rubus*, a relative difference of 58 %. An overall test on the effect of incubation environment (ambient incubation vs. incubation in the treatment plots) on litter mass loss showed that, after 2 years, there was no incubation effect (29.6 ± 0.7 vs. 29.2 ± 0.6 %, mean ± SE mass loss), but after 4 years, mass loss in the treatment plots was significantly (*P* < 0.02) higher (49.6 ± 1.5 % vs. 45.1 ± 1.2 %). Next, we tested for each species separately, both after 2 and after 4 years of incubation, whether the incubation environment (ambient vs. the treatment plot) had a significant positive effect on litter mass loss. This effect was only significant (*P* < 0.003) for *Betula* litter after 4 years of incubation, where overall mass loss was increased from 43.4 ± 1.6 % to 51.0 ± 2.1 %.

**Table 4:** Results of 3-way ANOVAs for mass loss data after 2 and 4 years in ambient and treatment plots as dependent on species, summer and winter treatments (see Table 1)

Incubation environment	Ambient		Treatment	
	Mass loss 2 years	Mass loss 4 years	Mass loss 2 years	Mass loss 4 years
Species (*df* = 2)	60.9****	13.9****	34.3****	11.1****
Summer treatment (*df* = 1)	0.6	0.7	0.2	0.1
Winter treatment (*df* = 2)	6.6**	0.9	2.2	2.2
Species × summer (*df* = 2)	0.2	3.2*	7.3***	0.8
Species × winter (*df* = 4)	0.7	1.5	0.4	2.1
Summer × winter (*df* = 2)	0.5	0.8	0.7	1.6
Species × summer × winter (*df* = 4)	1.8	1.3	0.7	1.1

The *F* values for the main effects and their interactions are presented, together with their level of significance

* *P* < 0.05, ** *P* < 0.01, *** *P* < 0.001, **** *P* < 0.0001

Despite the significant effects of the treatments on initial litter chemistry (Fig. 1; Tables 2 and 3), there were only a few and relatively minor (compared to the interspecific differences) effects of litter treatment origin on litter mass loss in the ambient incubation environment (Fig. 2; Table 4). There was only a significant effect of the winter treatments on 2-year mass loss. At the species level, this was expressed by higher (29.8 vs. 28.4 %) 2-year mass loss of *Betula* in response to the winter treatment and higher (32.6 vs. 31.6 %) mass loss of *Rubus* in response to the spring treatment (which was included in the winter treatments in the overall analysis of Table 4). The significant species × summer interaction points to species-specific responses to summer warming in the ambient incubation environment after 4 years. This was due to the response of *Calamagrostis* for which litter from summer warming treatments showed reduced litter mass loss compared to the summer ambient treatments (34.6 vs. 43.5 %), whereas the other species showed no significant response (Fig. 2; Table 5).

**Table 5:** Results of 3-way ANOVAs for mass loss data after 2 and 4 years in ambient and treatment plots as dependent on summer, winter and spring treatments (see Table 1) (*df* = 1)

Incubation environment	Ambient		Treatment	
	Mass loss 2 years	Mass loss 4 years	Mass loss 2 years	Mass loss 4 years
*Calamagrostis lapponica*	**23.3** **±** **0.6**	**38.4** **±** **2.0**	**23.8** **±** **0.9**	**40.7** **±** **3.0**
Summer treatment	0.1	4.4*	13.5***	0.5
Winter treatment	0.7	2.1	1.4	3.4
Spring treatment	1.9	1.3	2.5	1.1
*Betula nana*	**31.6** **±** **1.5**	**43.4** **±** **1.6**	**31.4** **±** **1.1**	**51.0** **±** **2.1**
Summer treatment	0.3	0.8	4.5*	0.1
Winter treatment	6.3*	0.2	0.2	2.9
Spring treatment	2.9	0.3	2.6	6.9*
*Rubus chamaemorus*	**33.5** **±** **0.7**	**52.4** **±** **1.5**	**33.7** **±** **0.9**	**55.8** **±** **2.1**
Summer treatment	0.5	0.1	0.1	1.0
Winter treatment	2.3	0.7	0.2	0.2
Spring treatment	6.4*	1.1	0.9	1.3

The *F* values for the main effects are presented, together with their level of significance. Error *df* = 26. Numbers in bold present the overall (averaged over all treatments) mean ± SE % mass loss

* *P* < 0.05, *** *P* < 0.001

Overall, there were no main treatment effects on mass loss in the treatment incubation environment (Table 4). However, there was a significant species × summer interaction for 2-year mass loss. This was due to the response of *Calamagrostis* for which summer warming reduced litter mass loss from 26.5 to 21.1 % in the treatment plots after 2 years, whereas for *Betula* 2-year mass loss was increased from 29.3 to 33.4 % by the summer treatment (Fig. 2; Table 5). After 4 years, spring warming reduced litter mass loss of *Betula* from 54.5 to 45.5 %.

### Nutrient dynamics

**Fig. 3:**
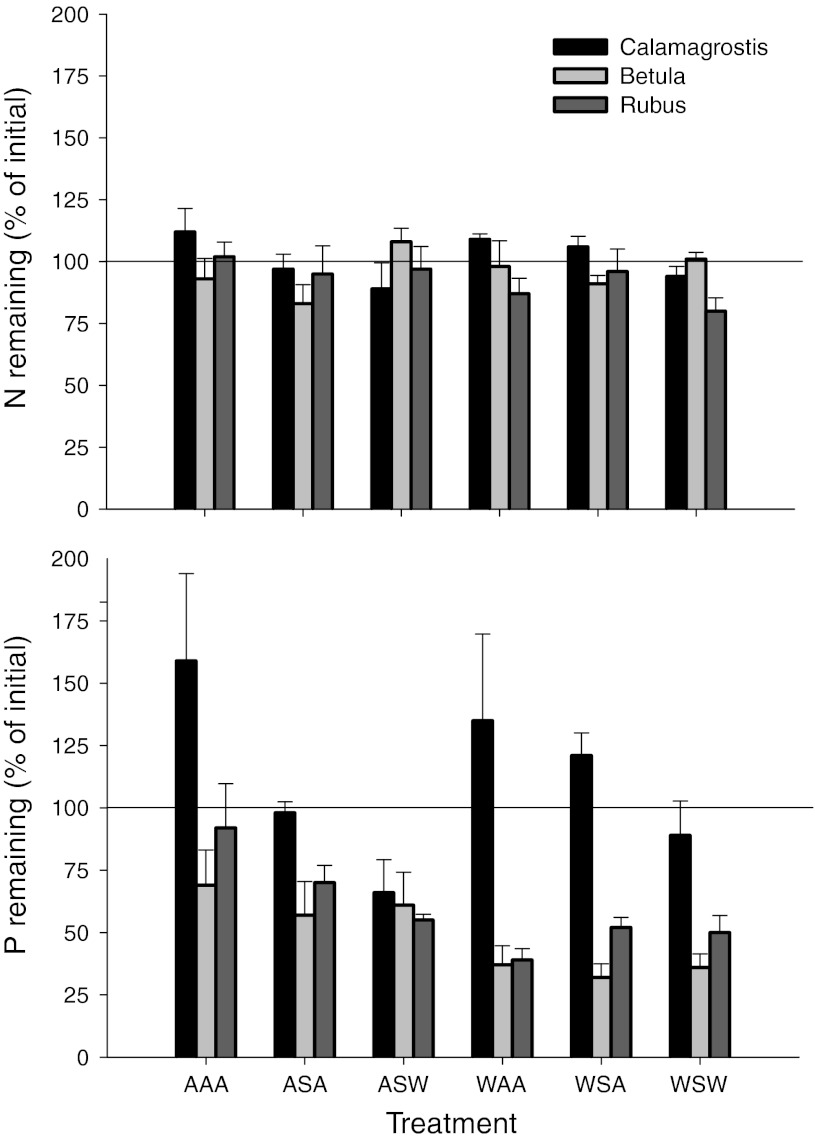
Amounts of N and P remaining (as % of initial amount) in leaf litter of three sub-arctic bog species after 4 years of incubation in the treatment plots in relation to climate treatments (see Table 1). Data are means ± SE (*n* = 5). The *horizontal line* is the 100 % line (no net change). Values >100 % indicate net immobilization and values <100 % net mineralization

An overall test on the effect of incubation environment (ambient incubation vs. incubation in the treatment plots) on the amount of N remaining showed neither an effect of incubation environment nor differences among species or treatments (Fig. 3; Table 2). Remarkably, all values were around 100 % of the initial amount, indicating that, during these 4 years, no net N mineralization had occurred in any litter type and treatment in spite of substantial mass losses ranging broadly between 40 and 60 %. Also, within each incubation environment separately, there were no significant effects of species or treatment. However, an analysis for individual species showed that spring warming increased N release from the litter of *Betula* (Table 3; Fig. 3).

Also, for P, there was no effect of the incubation environment on the amount of remaining P in the litter. However, in contrast with N, the amount of remaining P after 4 years differed strongly among species and treatments and showed an overall reduction in response to summer warming and the winter treatments (Fig. 3; Table 2). The significant species × summer and species × winter interactions indicate that the responses were idiosyncratic. Even after 4 years, *Calamagrostis* litter still had net P immobilization in most treatments, but spring warming (ASW treatment) enhanced net P release as evidenced by a reduction in the amount of remaining P (Fig. 3; Table 3). In *Betula*, there was net P release in all treatments, and this was stimulated by the summer treatments (Fig. 3; Table 3). In *Rubus*, net P release occurred in all treatments but the control. For this species, net P release was also increased by summer warming (Fig. 3; Table 3).

## Discussion

### Interspecific variation rather than direct or indirect species’ responses to climate treatments contribute to differences in litter decomposition rates

In line with our first hypothesis, the observed moderate changes in litter chemistry in response to the treatments hardly affected litter decomposition rates, as is shown by the relatively low response to the treatments in the ambient incubation environment. The few responses that were found were minor in effect size. For example summer warming reduced litter N concentrations in *Rubus* by about 25 %, whereas litter mass loss was not significantly reduced (Figs. 1 and 2). These observations are in agreement with earlier studies that found that experimentally induced changes in litter chemistry result in unchanged, or only slightly changed, litter decomposition rates, most likely because the secondary litter chemistry is not very responsive to environmental change, in contrast with the macro-nutrient concentrations (e.g., Hobbie and Vitousek 2000; Aerts et al. 2003, 2006a; Vivanco and Austin 2008). These results suggest that the relative constancy of the constitutive (secondary) litter chemistry of plant species (‘species identity’) outweighs the effects of experimentally imposed changes in the more responsive litter chemistry traits, such as N and P concentrations.

The relatively low responsiveness of litter decomposition (compared to the interspecific differences; cf. Table 5) to the changes in litter chemistry caused by the experimental treatments was sustained in the treatment incubation environments, even after 4 years. This implies that the moderate climate change scenarios that we experimentally imposed only have a minor impact on litter decomposition, at least for the duration of this study (4 years). At longer timescales, this might change (cf. Moore et al. 2011), but at this peatland site longer studies are impossible as the *Sphagnum* mosses invade the litter bags more and more, making it impossible to accurately determine remaining mass. For the summer warming treatments, the limited response to the treatments is in line with the meta-analysis of experimental warming studies in cold biomes (34 site-species combinations), generally involving a 1–1.5 °C increase in summer temperature, showing that warming resulted in only slightly increased decomposition rates (Aerts 2006). In other studies, however, where summer temperature increases were much larger (>4 °C), a substantial increase of decomposition rates was found (e.g., Hobbie 1996; Cornelissen et al. 2007). Thus, the relatively low responsiveness that we found does not reflect insensitivity of the decomposer sub-system to increased temperatures, but just a moderate response to a moderate temperature increase. This implies that, for the coming decades, in which temperatures in these sub-arctic regions will only moderately increase (IPCC 2007), only slight increases in litter mass loss may be expected. However, by the end of this century, when temperatures might have gone up by 4–7 °C (IPCC 2007), more profound changes may be expected. In addition to that, we found in an earlier study performed in this experiment (Dorrepaal et al. 2009) that old soil organic matter in these ecosystems is very sensitive to slight temperature changes. This differential response of fresh litter and old SOM to increased temperatures warrants further study.

The largest source of variation in litter decomposition was species identity as shown by the very substantial differences in decomposition rates among species (cf. Quested et al. 2003; Dorrepaal et al. 2005; Cornwell et al. 2008). Thus, the changes in the species composition and structure of plant communities, which have been observed in medium-term warming studies in cold biomes (cf. Walker et al. 2006), will have much more impact on litter decomposition than climate change-induced phenotypic responses.

### Seasonal climate manipulations do not affect litter N mineralization, but stimulate P release

Contrary to our second hypothesis, we found, even after 4 years of incubation, no or hardly any net litter N mineralization in any of the treatments. We also observed this very slow net N release from sub-arctic leaf litters in the controls of a nearby dry tundra site for *Betula* and *Rubus* (*Calamagrosti*s was not included in that study) (Aerts et al. 2006a). A possible explanation is provided by Rinnan et al. (2007), who studied the responses of the microbial community in a sub-arctic heath, close to our research site, to experimental warming. They found that the microbial community immobilized substantial amounts of N, and they suggested that the microbial community probably withdraws substantial amounts of nutrients from the inorganic plant-available pool. This is supported by recent data obtained in our study site, where we found that most of the N is occluded in organic and microbial N, but hardly in inorganic N (Weedon et al. 2012). This slow N release is in line with the global scale litter N release study of Parton et al. (2007), who found that in tundra ecosystems N release from non-indigenous standard litters with initial N concentrations of 0.6 % or lower (such as in *Calamagrostis* and *Betula*; Fig. 1) takes more than 6 years. However, for litters with an initial N concentration of 0.8 % (such as *Rubus*), it would, according to that study, only take 2.6 years, which is clearly not the case in our study. This emphasizes that characterizing litters by their initial N concentration alone ignores the fact that the litter traits that control litter decomposition rates do not all vary in a similar fashion when comparing different plant species (cf. Fréschet et al. 2011).

At first sight, the lack of response of N release to the experimental treatments seems to contradict the observed stimulation of net N mineralization in response to warming in many high-latitude sites (e.g., Schmidt et al. 1999; Rustad et al. 2001; Aerts et al. 2006b; Rinnan et al. 2007). However, it should be noticed that those studies refer to effects on mineralization of the total soil profile (or at least the upper 10 cm, where most of the organic material is situated), and that many of these studies involve experiments where the climate warming treatments have been applied for periods of up to 10 years. As a result, there may have been substantial accumulation of litter formed during the experimental treatments, and part of that litter may have passed the period of net immobilization and gone into the net N mineralization phase. According to our results, and to the results of Parton et al. (2007) for litters with very low initial N (<0.6 %), this takes more than 4 years.

Phosphorus dynamics of the decomposing litter was completely different compared to N. First of all, there were substantial interspecific differences in litter P dynamics: *Calamagrostis*, the species with a very low initial litter P concentration, showed net P immobilization in most treatments, but spring warming resulted in net P release. For the other two species, summer warming resulted in substantial net P release. This was especially the case for *Betula*, because summer warming not only increased initial litter P concentrations strongly but also stimulated the relative P release (expressed as % of the initial amount), as a result of which the flux of P into the soil increased substantially. Thus, especially summer warming considerably speeds up P cycling. The relative lack of P mineralization for *Calamagrostis* is consistent with the high C/P ratio in the initial litters (Fig. 1), whereas the evidence for considerable P mineralization in *Betula* and *Rubus*, whose initial C/P ratio falls in the range of 800–1,000, is close to the value at which P mineralization occurs (Moore et al. 2011). The results of Moore et al. (2011) that P mineralization in decomposing litters is more affected by environmental controls than N mineralization, and the observed idiosyncratic P interactions, support this.

Our results contrast with a study conducted by Rinnan et al. (2007) in a nearby mesic dwarf shrub/graminoid heath where summer warming had no effect on soil P mineralization. However, in that study, mineralization of the upper 5 cm of the soil was studied, thereby including both fresh litter and older SOM. Potential responses of the litter may thereby have been masked by a lack of response of the bulk soil.

Due to these differential responses of N and P mineralization to the treatments, the already existing N-limitation of plant growth in this sub-arctic peat bog may be sustained and probably further reinforced by climatic change. At the start of this decomposition experiment, N/P mass ratios in mature leaves of the dominant species varied between 13 and 14 (Aerts et al. 2009), indicating N-limited plant growth (Güsewell 2004). It is to be expected that N/P mass ratios will decrease in response to the climatic treatments, resulting in a stronger N-limitation of plant growth in this bog. However, it should be realized that these observations refer to plant litter only and not to soil organic matter that was formed prior to the start of the experimental manipulations. Recent measurements in our experiment showed that summer warming increases both N mineralization from soil organic matter, and also the flux of extractable organic N, which is about an order of magnitude higher than the mineral N flux (Weedon et al. 2012). The presence and type of mycorrhizal infection determine whether plant species have access to these organic N sources (Kielland 1994; Michelsen et al. 1998). Mycorrhizal species have access to organic N sources, whereas the non-mycorrhizal and arbuscular mycorrhizal (AM) species mainly assimilate inorganic N sources. Of the three species under study, *Betula* is ectomycorrhizal, whereas both *Calamagrostis* and *Rubus* are non-mycorrhizal (Michelsen et al. 1996, 1998). These growth form differences in soil N use have implications for the type of nutrient-limitation they experience, and are important determinants of the competitive ability of these species under conditions of N-limited growth (Aerts 2002).

In conclusion, our results show that moderate changes in summer temperatures and/or winter snow addition have only limited effect on litter decomposition rates and N dynamics, but summer warming and, to a lesser extent spring warming, do stimulate litter P release. As a result, N-limitation of plant growth in this sub-arctic bog may be sustained or even further promoted. However, the extent of N-limitation will depend greatly on the relative abundance of key vascular species with their different chemical compositions and type of mycorrhizal associations.
